# Patient Engagement and Attitudes Toward Using the Electronic Medical Record for Medical Research: The 2015 Greater Plains Collaborative Health and Medical Research Family Survey

**DOI:** 10.2196/11148

**Published:** 2019-03-12

**Authors:** Ann M Davis, Lawrence P Hanrahan, Alex F Bokov, Sarah Schlachter, Helena H Laroche, Lemuel Russell Waitman, GPC Height Weight Research Team

**Affiliations:** 1 University of Kansas Medical Center Kansas City, KS United States; 2 University of Wisconsin Madison Madison, WI United States; 3 University of Texas Health Science Center at San Antonio San Antonio, TX United States; 4 Children's Mercy Kansas City Kansas City, MO United States; 5 University of Iowa Iowa City, IA United States

**Keywords:** electronic health record research, survey, caregivers, engagement

## Abstract

**Background:**

Electronic health records (EHRs) are ubiquitous. Yet little is known about the use of EHRs for prospective research purposes, and even less is known about patient perspectives regarding the use of their EHR for research.

**Objective:**

This paper reports results from the initial obesity project from the Greater Plains Collaborative that is part of the Patient-Centered Outcomes Research Institute’s National Patient-Centered Clinical Research Network (PCORNet). The purpose of the project was to (1) assess the ability to recruit samples of adults of child-rearing age using the EHR; (2) prospectively assess the willingness of adults of child-rearing age to participate in research, and their willingness (if parents) to have their children participate in medical research; and (3) to assess their views regarding the use of their EHRs for research.

**Methods:**

The EHRs of 10 Midwestern academic medical centers were used to select patients. Patients completed a survey that was designed to assess patient willingness to participate in research and their thoughts about the use of their EHR data for research. The survey included questions regarding interest in medical research, as well as basic demographic and health information. A variety of contact methods were used.

**Results:**

A cohort of 54,269 patients was created, and 3139 (5.78%) patients responded. Completers were more likely to be female (53.84%) and white (85.84%). These and other factors differed significantly by site. Respondents were overwhelmingly positive (83.9%) about using EHRs for research.

**Conclusions:**

EHRs are an important resource for engaging patients in research, and our respondents concurred. The primary limitation of this work was a very low response rate, which varied by the method of contact, geographic location, and respondent characteristics. The primary strength of this work was the ability to ascertain the clinically observed characteristics of nonrespondents and respondents to determine factors that may contribute to participation, and to allow for the derivation of reliable study estimates for weighting responses and oversampling of difficult-to-reach subpopulations. These data suggest that EHRs are a promising new and effective tool for patient-engaged health research.

## Introduction

In 2013, The Greater Plains Collaborative (GPC) was established as a Clinical Data Research Network (CDRN), funded by the Patient-Centered Outcomes Research Institute to securely collect and organize patient health information obtained during routine care in its member health systems [1]. To date, 13 such CDRNs have been funded, creating a national “network of networks.” These networks are organized by a coordinating center and overseen by the National Patient-Centered Clinical Research Network (PCORnet) [2]. The purpose of CDRNs and PCORnet is to support efficient clinical research by creating centralized access to the deidentified data of millions of patients across the country. Each CDRN is responsible for harmonizing patient data across its member systems, and for creating streamlined governance and procedures to facilitate researcher access. Importantly, CDRNs actively involve a variety of stakeholders, including patients, clinicians, health care system leaders, and other stakeholders, to build and oversee CDRN activities. To test each CDRN’s ability to identify and recruit patients with a particular condition, and to test the ability to harmonize data elements within a network, each CDRN was required to create three cohorts: one of a common disease [3], one of a rare disease (amyotrophic lateral sclerosis [in progress]), and one concerning height and weight; the GPC height and weight cohort is the one described herein.

### Greater Plains Collaborative Member Sites

In phase 1 of funding, the GPC consisted of 10 health systems, with the data of approximately 6 million people across seven states, north and south across the Great Plains region. Member institutions included Children’s Mercy Hospital (Kansas City, MO), University of Kansas Medical Center, Marshfield Clinic, Medical College of Wisconsin, University of Iowa Healthcare, University of Minnesota, University of Nebraska Medical Center, University of Texas Health Science Center at San Antonio, University of Texas Southwestern Medical Center, and University of Wisconsin-Madison.

Covering more than 1300 miles, the broad reach of the GPC network encompasses large swaths of rural populations as well as multiple urban centers. Four systems in the GPC have established significant relationships with Native American populations. Two health systems located in Texas, the University of Texas Southwestern Medical Center and the University of Texas Health Science Center at San Antonio, serve large Hispanic populations. Of the 10 member health systems participating in the Height Weight Cohort’s Health and Medical Research Family Survey (HMRFS), all provide comprehensive adult and pediatric care, with the exception of Children’s Mercy Kansas City, which exclusively serves children. In phase 2 of the GPC, two additional members were added—University of Missouri and Indiana University—but the data reported here predate their participation.

### Health and Medical Research Family Survey

The purpose of the HMRFS was to conduct a demonstration survey (Textbox 1) across all participating GPC sites focused on the topic of pediatric height and weight, and specifically, pediatric overweight and obesity. The expected outcome of the project was to understand the practical challenges and operational details of a large, semi-interconnected system such as the GPC for conducting collaborative prospective data collection-based research focused on pediatric obesity. Aims of the project were to (1) estimate the willingness of individuals to be contacted about research activities, and their response rate; (2) obtain information on the attitudes of parents and adults of child bearing age about research, including participation of their child/ren; (3) gain insight into participant attitudes about the use of gathered data for both local and national research; (4) explore the impact of various demographic factors on survey outcomes and survey response rate; (5) examine if there are differences between individuals across various weight classes; and (6) determine if there are regional variations in all of these. Although previous studies have been published on adult obesity using a CDRN funded by the Patient-Centered Outcomes Research Institute [4], these studies were retrospective in nature and reported on the number of patients in the network who met certain criteria. In contrast, the HMRFS of the GPC not only gathered retrospective data on individuals who met certain specific inclusion criteria but also conducted prospective data collection by containing a random group of individuals from the subsample at each site with a survey invitation. The goal for all participating sites was to contact at least 1000 individuals or a number deemed sufficient to garner at least 100 complete responses per site to the survey.

**The Health and Medical Research Family Survey.**

1. Have you or anyone in your family ever been a participant in any type of medical research?

2. Can medical researchers contact you to tell you about opportunities for you or someone in your family to participate in a medical research project?

2B. Please select any of the answers that describe what might help you decide to be contacted. You may choose one or more than one answer if you like: it depends on what the research is about; it depends on how much time it would take; it depends on whether my doctor thinks that it would be a good idea; it depends on whether I would be paid; it depends on whether it would involve just me or whether it would involve my child or children; it depends on something else.

3. Do you have a child or children under the age of 21?

4. If you have a child or children, would you be willing to be contacted about opportunities for your child or children to take part in a medical research project? You may choose one or more than one answer if you like: it depends on what the research is about; I would be interested if the research is about; it depends on how much time it would take; it depends on whether my doctor thinks that it would be a good idea; it depends on whether I would be paid; it depends on whether it would involve just me or whether it would involve my child or children; it depends on something else.

5. Would you be willing to talk to family members or friends about taking part in a medical research study?

6. The information your doctor collects about you is very important. When researchers combine health information obtained from many people, it can help find ways to improve health. It can also tell researchers which treatments work best for different people. How do you feel about *your medical information* being used for research?

7. The information your doctor collects about you is stored on computers. People have to have permission to look at or share your electronic health information. It is possible to remove personal information (like your name, birth date, etc) before it is shared. This process is called “deidentification.” *If your health care provider deidentified your health information,* how would you feel about your information being shared?

Will you please answer the following questions about yourself, and (if appropriate) about your child.

8. How tall are you? Please write that information in the blanks below.

9. What is your approximate weight?

10. Do YOU have any of the following conditions? High blood pressure (also called hypertension), high cholesterol, high triglycerides or hyperlipidemia, high blood sugar or diabetes, cancer (any type)?

11. Do any of the following blood relatives (your biological father, mother, brother, sister, uncle or aunt, son or daughter) have any of the following conditions? High blood pressure (also called hypertension), high cholesterol, high triglycerides or hyperlipidemia, high blood sugar or diabetes, cancer (any type)?

## Methods

The Height Weight Cohort team, which consisted of representatives from all sites, began regular meetings in January 2014. Based on discussions and collective interest, the group quickly decided to develop its cohort and survey around a pediatric population. Weekly working group calls established an interest in characterizing the cohort around data elements that would be attainable for the nascent GPC network and creating a survey that could be used as a building block for future GPC and healthy weight cohort work (manuscripts in preparation). Thus, the HMRFS focused on respondents’ willingness to take part in future clinical research as well as key demographic and health-related issues theorized to impact these responses.

### Institutional Review Board Process

Through its efforts to streamline governance, the GPC Institutional Review Board (IRB) Consortium was established to facilitate IRB review and approval. The consortium, including all the GPC sites, signed a common IRB reliance agreement and adopted standard operating procedures which would govern the reliance process. The University of Texas Health Science Center at San Antonio served as the reviewing IRB site for the HMRFS across the GPC network. The HMRFS team of investigators and staff developed the necessary IRB documents, which were submitted to the IRB at the University of Texas Health Science Center at San Antonio. Once the documents were reviewed and approved by the reviewing IRB, the documents were shared with all other participating sites’ IRBs, and these documents were approved under the existing overall IRB reliance agreement. The GPC went on to be an early adopter of the SMART IRB platform, which now has more than 175 other participating institutions and is designed to facilitate multisite research and implement the NIH Single IRB Review Policy.

### Data Harmonization

Data were extracted from an open source data warehouse platform called Integrating Informatics from Bench to Bedside (i2b2). Each of the participating sites had an i2b2 instance deployed, where a deidentified version of all structured data from their respective electronic health record (EHR) systems was stored. As a result, no site had to transmit any identifying information to any other site. The other required component was Research Electronic Data Capture (REDCap). REDCap is noncommercial software developed at Vanderbilt University for the purposes of conveniently capturing research data, including surveys. All participating sites hosted the online survey on their respective local REDCap servers. At sites where paper and telephone responses were accepted, survey personnel manually filled in REDCap surveys on behalf of the respondents. The survey data and the EHR data extracted from i2b2 were merged using a nonidentifying index. Data were extracted from the EHR using DataBuilder collated into an analyzable tabular form using DataFinisher.

**Table 1:** Detailed list of site, adult/pediatric cohort, and contact method.

Site	Cohort makeup	Contact method^a^
Children’s Mercy Hospital	Pediatric	Email
University of Kansas Medical Center	Pediatric	Email
Marshfield Clinic	Pediatric	Email
Medical College of Wisconsin	Adult	Email
University of Iowa Healthcare	Pediatric	USPS
University of Minnesota	Pediatric	USPS
University of Nebraska Medical Center	Adult	Email
University of Texas Health Science Center at San Antonio	Pediatric	USPS
University of Texas Southwestern Medical Center	Pediatric	Email
University of Wisconsin-Madison	Adult and pediatric	Portal

^a^Email: electronic mail on file; portal: patient portal feature of the electronic medical record system; USPS: United States Postal Service.

### Cohort Identification

Cohort selection was completed using the i2b2 web client [5]. The inclusion criteria were manually translated at each site to the equivalent local codes. Each site then ran the resulting query, and their local i2b2 server generated a “patient set”—a list of deidentified patient numbers. Each site’s informatics team had a crosswalk file matching the deidentified patient numbers to actual medical record numbers or database keys for their local patients. Neither were shared externally. The local informatics teams would use these identifiers to obtain names and contact information from the local EHR system, and these contact lists were securely transmitted to the respective local HMRFS site leads, who used them for recruitment. Also, information regarding race, ethnicity, and class of insurance provider (eg, private, Medicaid, employer, government, self-pay) was gathered directly from each site’s EHR. Income was gathered through median household income for the census block group in which each patient’s address was located, as obtained from the 2013 American Community Survey census block group data (tables B19013 and B19013A-I) [6].

### Participants

Using the EHR at each site, a list was developed of all individuals who met the inclusion and exclusion criteria for the study. Most sites targeted pediatric patients, but there were two sites for whom there were not sufficient pediatric patients available, so adult patients were targeted, and one site chose to target both pediatric and adult patients (see Table 1). For the pediatric patients, inclusion criteria included individuals who had at least one outpatient visit to the institution within the previous 36 months, with an age between 2 and 20 years, with both height and weight obtained at the same visit on at least one occasion, male or female gender, an address or email available in the EHR, a body mass index (BMI) over the fifth percentile for age and gender, as well as an identifiable parent or guardian. For the adult patients, some criteria were modified, and the criteria for an identifiable parent or guardian was removed. Specifically, age was modified to be between 21 and 49 years, and a BMI of 18.5 kg/m^2^ or higher. After the list of patients who met inclusion criteria was developed, deceased patients were removed (exclusion criteria) as well as cases with nonsensical heights, weights, and BMIs. Pregnant adult females were also removed, as were duplicates, resulting in a finalized patient list.

Potential participants were contacted through one of three means: the United States Postal Service (USPS), email, or through the patient portal feature of the electronic medical record systems. The method of contact was selected by each HMRFS site principal investigator based on logistics and local policy requirements. For a detailed list of sites and contact methods, see Table 1.

### Analysis Plan

Descriptive statistics were obtained for the overall cohort population, as well as the survey respondents. Univariate logistic regression models were fit to these observations as an exploratory screen in preparation for further analysis. Categorical variables with more than two levels were split into individual indicator variables to determine which levels correlated with increased participation. Predictor variables included patient demographics (age, race/ethnicity, BMI, income, insurance), recruitment type (USPS, email, EHR patient portal), and site. Finally, counts and percentages are reported for respondent attitudes toward research participation.

## Results

### Response Rates and Cohort Characteristics of Different Contact Methods

**Figure 1:**
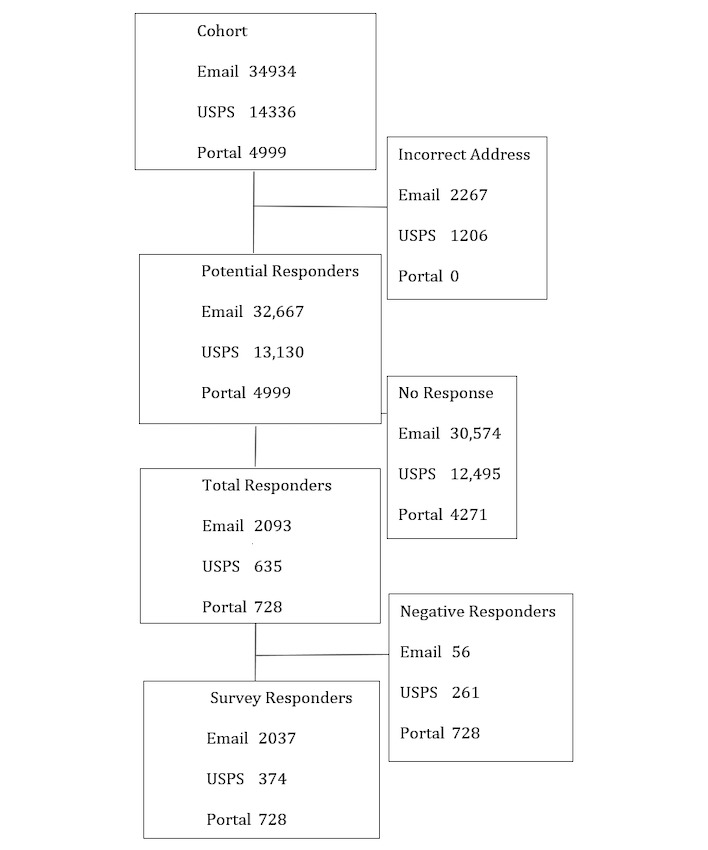
CONSORT diagram including all sites.

The final cohort included 54,269 individuals who met the inclusion and exclusion criteria (Figure 1). Of these, 34,934 (64.37%) were contacted via email, 14,336 (26.42%) were contacted via USPS, and 4999 (9.21%) were contacted via the portal in their site’s electronic medical record with an invitation to participate (Table 2). Of these, 3473 (6.39%) were identified to have incorrect contact information.

**Table 2:** Response rate by recruitment method across all sites.

Contact method	Cohort, n	Responders, n (%)
Email	34,934	2051 (5.55)
USPS	14,336	460 (3.11)
Portal	4999	728 (12.71)
Total	54,269	3239 (5.63)

No response was received from 47,340 of 54,269 (87.23%) individuals. This resulted in 3456 (6.37%) total responders, with 217 (6.26%) of these responding to indicate they wanted no future contact, and 100 (2.89%) responding only to the single question declining participation. A final sample of 3139 individuals were survey responders.

The index patient varied (adult or child) by site (Table 1), but cohort demographics are available individually for each site from the research team. Sample size by site varied from a minimum of 3411 (Children’s Mercy Hospital) to a maximum of 9849 (University of Nebraska Medical Center). Among the entire cohort sampled, the proportion of males at each site varied significantly (35.8%, 3523/9849/to 55.3%, 2393/4328) as did the racial makeup of the site sample, although all sites were predominantly white and non-Hispanic. Insurance status also varied significantly by site; the most common were self-pay (53.25%, 3195/6000), Medicaid (38.93%,1685/4328), and private insurance (88.10%4398/4992). Estimated median household income ranged from US$42,770 to US$67,020. The mean age of the index patient (child) ranged from 9.47 to 14.83 years. As noted previously, three sites recruited adults because they did not have enough pediatric patients to meet accrual targets. The mean ages of their patients ranged from 35.37 to 47.82 years, so they were asked the same questions about children residing at home and participating in research as were asked of the other sites. Finally, BMI category for the index patient varied significantly by site, with values ranging from 15.8% to 45.7% for overweight and 15.1% to 43.9% for obese.

### Results From Responders to the Health and Medical Research Family Survey

A total of 3139 responders completed the HMRFS across all sites (Table 3). Gender differed significantly by site, ranging from 25.3% (91/360) to 58.0% (47/81) male, as did race, ranging from 65.8% (75/115) to 95.5% (386/404) white. The percentage of Hispanic responders also varied significantly, with one site reporting a rate as high as 25.2% (29/115). Annual income ranged from US$45,000 to US$75,600. Age of index patient (child) ranged from mean 13.90 to mean 9.68 years, and age of the index patient (adult) ranged from mean 37.12 to 48.26 years. BMI category also varied significantly, for both overweight (11.6%, 11/95 to 51.9%, 42/81) and obese (12.9%, 51/396 to 44.5%, 289/728).

Responses indicate that across all sites, 29.44% (924/3139) of respondents (or their family members) had participated previously in medical research. Respondents were generally open to investigators contacting them about possible participation in studies (yes: 42.47%, 1333/3139; maybe: 38.71%, 1215/3139). Key factors in making the decision to participate in medical research included: topic of research (62.98%, 1977/3139), time (48.20%, 1513/3139), doctor recommendation (22.05%, 692/3139), reimbursement (17.23%, 541/3139), and child involvement (23.89%, 750/3139; Table 4). Most participants were in favor of their medical information being used for research (fantastic idea: 34.88%, 1095/3139; good idea: 48.01%, 1507/3139) with similar responses if the medical research was deidentified (fantastic idea: 31.98%, 1004/3139; good idea: 48.84%, 1533/3139).

The respondents who had children (2001/3139, 63.74%) also reported feeling generally positive about possibly allowing their child to take part in medical research (yes: 27.2%, 542/2001; maybe: 40.0%, 797/2001). Key factors in making the decision to allow their child to participate in medical research included topic of research (56.6%, 1132/2001), time (38.5%, 771/2001), doctor recommendation (26.4%, 529/2001), reimbursement (12.3%, 247/2001), and child involvement (13.7%, 275/2001; Table 5).

**Table 3:** Demographics of the sample and responders by recruitment method.

Demographics			Email	USPS	Portal	Total	*P* value
**Sample**							
	Total, N		34,934	14,336	4999	54,269	
	Responded, n (%)		2037 (5.8)	374 (2.6)	728 (14.6)	3139 (5.8)	
**Gender (male)**							<.05
	Total, n		15,947	7397	1825	25,169	
	Responded, n (%)		905 (5.7)	189 (2.6)	255 (14.0)	1349 (5.4)	
**Race**							<.05
	**White**						
		Total, n	25,708	10,208	4518	40,434	
		Responded, n (%)	1766 (6.9)	307 (3.0)	680 (15.1)	2753 (6.8)	
	**African American**						
		Total, n	3344	985	108	4437	
		Responded, n (%)	57 (1.7)	4 (0.4)	11 (10.2)	72 (1.6)	
	**Native American**						
		Total, n	160	69	21	250	
		Responded, n (%)	6 (3.8)	1 (1.4)	4 (19.0)	11 (4.4)	
	**Asian**						
		Total, n	860	442	94	1396	
		Responded, n (%)	38 (4.4)	7 (1.6)	17 (18.1)	62 (4.4)	
	**Other**						
		Total, n	2596	1951	5	4552	
		Responded, n (%)	97 (3.7)	41 (2.1)	1 (20.0)	139 (3.1)	
	**Unknown**						
		Total, n	1014	623	93	1730	
		Responded, n (%)	17 (1.7)	13 (2.1)	15 (16.1)	45 (2.6)	
**Ethnicity (Hispanic)**							<.05
	Total, n		3057	2139	94	5290	
	Responded, n (%)		68 (2.2)	34 (1.6)	16 (17.0)	118 (2.2)	
**Insurance status**							
	**Self-Pay**						<.05
		Total, n	5035	3190	2985	11,210	
		Responded, n (%)	343 (6.8)	99 (3.1)	433 (14.5)	875 (7.8)	
	**Medicare**						
		Total, n	573	3	442	1018	
		Responded, n (%)	16 (2.8)	0 (0.0)	71 (16.1)	87 (8.5)	
	**Medicaid**						
		Total, n	6672	2257	102	9031	
		Responded, n (%)	232 (3.5)	35 (1.6)	17 (16.7)	284 (3.1)	
	**Private insurance**						
		Total, n	16,393	7847	966	25,206	
		Responded, n (%)	1147 (7.0)	215 (2.7)	149 (15.4)	1511 (6.0)	
	**Other**						
		Total, n	1017	368	0	1385	
		Responded, n (%)	30 (2.9)	8 (2.2)	0 (0.0)	38 (2.7)	
	**Unknown**						
		Total, n	4073	613	344	5030	
		Responded, n (%)	215 (5.3)	16 (2.6)	58 (16.9)	289 (5.7)	
**BMI category**							
	**Overweight**						<.05
		Total, n	9159	4599	1349	15,107	
		Responded, n (%)	510 (5.6)	115 (2.5)	207 (15.3)	832 (5.5)	
	**Obese**						
		Total, n	10,372	3713	1893	15,978	
		Responded, n (%)	569 (5.5)	71 (1.9)	289 (15.3)	929 (5.8)	

**Table 4:** Adult respondents’ (adults of child-rearing age or parents of index child patients) thoughts regarding their participation in research (N=3139).

Survey question		n (%)
**1. Have you or anyone in your family ever been a participant in any type of medical research?**		
	Prefer not to answer	3 (0.01)
	No	1838 (58.55)
	Unsure	374 (11.91)
	Yes	924 (29.44)
**2. Can medical researchers contact you to tell you about opportunities for you or someone in your family to participate in a medical research project?**		
	Prefer not to answer	69 (2.19)
	No	504 (16.06)
	Maybe	1215 (38.71)
	Yes	1333 (42.47)
**2B. Please select any of the answers that describe what might help you decide to be contacted.**		
	What research is about	1977 (62.98)
	Specific topics	240 (7.65)
	How much time it would take	1513 (48.20)
	My doctor’s opinion	692 (22.05)
	Being paid	541 (17.23)
	Whether involves children	750 (23.89)
	Other	70 (2.23)
**3. Do you have a child or children under the age of 21?**		
	Prefer not to answer	13 (0.41)
	No	1106 (35.23)
	Yes	2001 (63.74)
**5. Would you be willing to talk to family members or friends about taking part in a medical research study?**		
	Prefer not to answer	35 (1.12)
	No	1102 (35.12)
	Unsure	967 (30.81)
	Yes	1000 (31.86)
**6. How do you feel about** ***your medical information*** **being used for research?**		
	Unsure	331 (10.55)
	Prefer no answer	23 (0.73)
	Not good idea	30 (0.95)
	Fantastic	1095 (34.88)
	Good idea	1507 (48.01)
**7. If your health care provider deidentified your health information, how would you feel about your information being shared?**		
	Unsure	379 (12.07)
	Prefer not to answer	21 (0.67)
	Fantastic	1004 (31.98)
	Good idea	1533 (48.84)
	Terrible	28 (0.89)
	Not good Idea	72 (2.29)
	Other	57 (1.82)
**10. Do you have hypertension?**		
	Prefer not to answer	9 (0.29)
	No	2479 (78.97)
	Unsure	64 (2.04)
	Yes	516 (16.44)
**10. Do you have high cholesterol?**		
	Prefer not to answer	11 (0.35)
	No	2281 (72.66)
	Unsure	159 (5.07)
	Yes	605 (19.27)
**10. Do you have diabetes?**		
	Prefer no answer	9 (0.29)
	No	2670 (87.93)
	Unsure	95 (3.03)
	Yes	276 (8.79)
**10. Do you have cancer?**		
	Unsure	80 (2.55)
	Prefer not to answer	13 (0.41)
	No	2691 (85.76)
	None	95 (2.89)
	Yes	150 (4.78)
**11. Do you have relatives with hypertension?**		
	Prefer not to answer	10 (0.32)
	No	888 (28.29)
	Unsure	200 (6.37)
	Yes	1967 (62.66)
**11. Do you have relatives with high cholesterol?**		
	Prefer no answer	11 (0.35)
	No	973 (30.99)
	Unsure	341 (10.86)
	Yes	1718 (54.73)
**11. Do you have relatives with diabetes?**		
	Prefer not to answer	10 (0.32)
	No	1488 (47.40)
	Unsure	188 (5.99)
	Yes	1344 (42.82)
**11. Do you have relatives with cancer?**		
	Unsure	140 (4.46)
	Prefer not to answer	12 (0.38)
	No	1388 (44.22)
	None	95 (3.03)
	Yes	1402 (44.66)

**Table 5:** Responses from caregivers with children regarding their child’s participation in research (n=2001).

Question		n (%)
**4. Would you be willing to be contacted about opportunities for your child or children to take part in a medical research project?**		
	Maybe	797 (39.83)
	No	613 (31.48)
	No children	4 (0.19)
	Prefer not to answer	36 (1.79)
	Yes	542 (27.09)
**4B. What helps you decide about children research?**		
	What research is about	1132 (56.57)
	Specific topics	133 (6.65)
	How much time it would take	771 (38.53)
	My doctor’s opinion	529 (26.44)
	Being paid	247 (12.34)
	Whether involves children	275 (13.74)
	Other	58 (2.89)

Looking at these responses by site (data available from study team), there was variability by site of more than 30% on some survey questions. For example, participation in previous medical research varied from 20.0% (35/174; University of Texas Health Science Center at San Antonio) to 68.4% (65/95; University of Texas Southwestern Medical Center). On the question of possibly being contacted for participation in future research, sites varied from a low of 39.5% (221/560; Medical College of Wisconsin ) to a high of 68.8% (65/95; University of Texas Southwestern Medical Center). Regarding having a child in the home, responses varied from 99.1% (232/235; Children’s Mercy Hospital, a pediatric hospital) to 30.9% (273/885; University of Wisconsin-Madison). The percentage of those with a child in the home who would allow that child to participate in medical research varied from 15.9% (57/360; University of Nebraska Medical Center) to 78.9% (75/95; University of Texas Southwestern Medical Center). Regarding the idea of their medical data being used in health research, favorable responses by site varied from a low of 78.0% (74/95; University of Texas Southwestern Medical Center) to a high of 92.6% (75/81; University of Minnesota). Finally, regarding the use of deidentified medical information being used in research, sites ranged from 79.3% (314/396; University of Kansas Medical Center) in favor to 90.1% in favor (73/81; University of Minnesota).

### Electronic Health Record Predictors of Participation

Prior to analysis, the 54,269 cohort members were randomly assigned to a development (n=10,751), validation (n=10,748), and test (n=32,770) subsets to avoid overfitting and bias due to within-sample testing. All analysis decisions were made based on the developmental subset. The first goal was to identify candidate predictors for survey participation from among the variables available for all members of the cohort (ie, those listed in Table 3). Accordingly, for each candidate predictor, a separate logistic regression model was fit to the developmental subset with responder status as the outcome. Discrete variables with multiple levels were broken up into an equal number of indicator variables. Significant predictors of increased response included increasing age, being white, and having insurance self-pay. Adult patient recruitment sites were associated with increased participation. Several factors predicted decreased participation, including Hispanic ethnicity, having Medicaid, being African American, and (at the site level) recruitment via USPS and recruitment of pediatric patients.

## Discussion

The purpose of the Height Weight Cohort’s HMRFS was to conduct a prospective demonstration survey across 10 participating sites in the GPC to (1) assess the ability to recruit samples of adults of child-rearing age using the EHR; (2) prospectively assess the willingness of adults of child-rearing age to participate in research, and (if parents) their willingness to have their children participate in medical research; and (3) to assess their views regarding the use of the EHRs for research.

### Recruitment of Parents and Adults of Child-Rearing Age Using the Electronic Health Record

These data suggest that the EHR can be used to recruit patients to medical research using the EHR portal, USPS, and email. The data indicate that the EHR portal obtained the most effective recruitment rate at 12.71%. Further analyses indicate that increasing age, being white, and having insurance self-pay predicted higher rates of participation, whereas Hispanic ethnicity, having Medicaid, and being African American predicted lower rates of participation. Regarding the recruitment method, recruitment via USPS predicted lower rates of participation, and the recruitment of pediatric patients also predicted lower rates of participation.

### Interest in Research Participation for Adults/Caregivers and Their Children

Survey data indicate that most respondents had not participated in research previously, but were maybe (38.9%) or definitely (42.7%) willing to be contacted for research participation. Caregivers were also interested in being contacted for research appropriate for their children, with 27.2% indicating yes and 40.9% indicating maybe. As a team of researchers constantly seeking research participants for our work, we are encouraged by these affirmative responses. Other data surveying adults about their willingness to participate in research have not indicated such high enthusiasm [7]; only 7.1% of respondents were willing to participate in weight-related research, but 82.2% were willing to participate in healthy lifestyles research. These data indicate that the topic of the research is a key factor in decision making, but specific topics were not assessed in the current study.

Survey respondents were also asked their opinions on whether medical information should be used for research, and an overwhelming majority responded positively (83.9%). Responses were equally positive when asked about the use of deidentified data (81.9%). Responses to the final two survey questions about the use of medical information (deidentified or non-deidentified) were overwhelmingly positive.

This study did have several strengths. First, unlike many other surveys, we were able to collect demographic information on the entire cohort that was invited to participate (also known as the sampling frame). This type of information is very helpful in determining how the respondent sample may have been biased in some way. Second, because the methodologies for patient contact were low burden (email, portal, USPS) we were able to contact a very large number of patients with little to no budgetary implications. Also, we are one of the first studies to use the electronic medical record portal to contact participants for research. This study did have several weaknesses. First, our overall survey response was low (6.2%). Other studies conducted through the CDRN using survey methodology indicated response rates of 3% to 6% using the same methodologies used here [8]. Therefore, although these rates are low, they are consistent with previously published literature in this area. Even so, it is possible that the individuals who responded to our survey had a positive attitude toward research, which predisposed them to respond to the survey, and could have influenced our positive survey findings. Second, due to inconsistencies across sites, we were unable to use a single method of contacting participants (some sites did not have emails on file, others did not permit the use of their EHR for research). Third, for pediatric patients, when an email was listed in their medical record, it was unclear in some cases whether this was the child email or the parent email, which required further follow-up and clarification. We are hopeful that as sites move toward more electronic communication with their patients, there will be fields for both parent email and child email when appropriate.

Moving forward, questions remain about how best to use the EHR to identify and contact patients. As we have shown, each contact method has its limitations. Traditional mail can be labor intensive and expensive. Email is currently limited by the lack of data in the EHR system, but this should improve over time. Using the EHR directly through the electronic portal is limited by concerns about intrusion and privacy at some sites, which may not allow such contact in their health systems. Of note, the site in our study that used the EHR portal was only able to obtain permission for the use of the portal after the patient advisory board advocated for the project. The other concern [9] is that certain populations (such as the elderly) may be less likely to use electronic media such as email or the EHR portal, and thus may be excluded from studies using these methods. Most previous research studies have used the EHR to identify individuals followed by mailed invites and phone calls. There are reports of trials using the patient portal who found it was helpful, and in one case better than other methods, but not sufficient to use alone. It was also better at reaching younger patients. A recent survey of the Clinical and Translational Science Award consortium found that only 20% of institutions had EHR patient portals that could notify patients about research opportunities. However, another 70% were exploring or planning to use such tools [9]. Trials may consider using a combination of electronic methods for the majority with traditional mail for a subsample, but future research is needed on this topic.

In conclusion, this study demonstrates that the linkage of data from EHRs at multiple institutions can be a useful tool to gather large study samples for research. This study was novel in that we were able to gather data without sharing patient information outside of the home institution, which may provide a helpful example for future researchers required to do so. Also, this study focused on caregiver responses regarding their children, a population that has not been included in other research regarding the use of EHRs for research. Further research into how to maximize these new research opportunities is warranted.

## Appendix

Multimedia Appendix 1 Description of GPC funding.

## Abbreviations

BMI: body mass index
CDRN: Clinical Data Research Network
EHR: electronic health record
GPC: Greater Plains Collaborative
HMRFS: Health and Medical Research Family Survey
i2b2: Integrating Informatics from Bench to Bedside
IRB: Institutional Review Board
USPS: United States Postal Service
