# Supplementary material for: Patient Engagement and Attitudes Toward Using the Electronic Medical Record for Medical Research: The 2015 Greater Plains Collaborative Health and Medical Research Family Survey
Source: JMIR Res Protoc. 2019 Mar 12;8(3):e11148. doi: 10.2196/11148 (PMC6434393; doi:10.2196/11148)
Supplement: Multimedia Appendix 1 [file resprot_v8i3e11148_app1.pdf]

Patient-Centered Outcomes Research Institute

The Greater Plains Collaborative (GPC)

Project Summary

The Greater Plains Collaborative (GPC) is a new network of 10 leading medical centers in seven states committed to a shared vision of improving healthcare delivery through ongoing learning, adoption of evidence-based practices, and active research dissemination. Partners by state are:

- Kansas, the University of Kansas Medical Center;
- Missouri, Children's Mercy Hospital;
- Iowa, University of Iowa Healthcare;
- Wisconsin, the University of Wisconsin-Madison, the Medical College of Wisconsin, and Marshfield Clinic; Minnesota, the University of Minnesota Academic Health Center; Nebraska, the University of Nebraska Medical Center;
- and Texas, the University of Texas Health Sciences Center at San Antonio and the University of Texas Southwestern Medical Center.

The GPC builds on strong research programs at our sites, existing community engagement, informatics infrastructures and data warehouses developed through the National Institutes of Health (NIH) Clinical and Translational Science Awards (CTSA) initiative at most of our sites, extensive expertise with commercial electronic health records (EHR) systems and terminology

standardization, and strong working relationships between investigators and healthcare system information technology departments. Our network brings together a diverse population of 6 million people across 1300 miles covering seven states with a combined area of 679,159 square miles, and that includes patients in underserved minority and rural areas (RC1). We complement considerable investments in EHRs by our healthcare systems with existing NIH-funded open source technology (e.g., i2b2, REDCap) to provide a cost-effective common data model that promotes data transparency and interoperability (RC2, RC3). In addition to our data repositories and their accompanying data security and de-identification methods (RC11), the GPC builds on other existing CTSA and research investments for:

1. patient and community engagement (RC4), which will be extended to provide patient and clinician voices and participation in all GPC activities;
2. biostatistics and epidemiologic expertise to inform research designs and informatics methods for research participant identification, recruitment (RC6), and collection of patient-reported outcomes (RC8);
3. human subjects protection, Institutional Review Board (IRB), and contract approvals (RC10) that span institutional boundaries and cross state lines; and
4. mature biospecimen management and unique resources at several of our institutions (RC12).

The GPC also has an innovative research methods core that will complement and inform our informatics team. Across our 10 sites, our informatics team has expertise in commercial EHR development and project management (RC13), clinical decision support, and standard terminology development. We will tailor our existing EHR research modules and patient portals and, where needed, will

integrate existing clinical research systems in order to support comparative effectiveness randomized trials within the clinical workflow (RC9) and to increase robust collection of patient-reported outcome measures.

Using input from community members and community advisory groups across our network, we selected breast cancer as our high-prevalence disorder. In 2013 alone it is estimated that 232,340 women will be diagnosed with breast cancer. Not only was breast cancer the top choice of the community members polled, but focusing on breast cancer will allow us to capitalize on our existing relations with National Cancer Institute (NCI) centers at GPC sites for engaging investigators and patients. Our network also has considerable strengths in neuromuscular rare diseases. After conferring with clinicians treating neuromuscular rare diseases in our GPC network, and the leadership of a neurological patient advocacy group, we selected amyotrophic lateral sclerosis (ALS) as the rare disease for which we could readily access all patients across our network. ALS has an estimated prevalence of 40-60 cases per 1,000,000 and an incidence of 0.4 to 1.8 per 100,000. As detailed in RC6, all ALS patients residing around our GPC sites are seen in the specialty clinics of our GPC neuromuscular rare disease specialists and thus we will have complete access to this population in our broad geographic region.

Fulfilling the PCORI vision of an engaged network of patients, investigators, and providers is fundamentally built on trust earned from successful relationships. Not only do we have strong community engagement programs across our sites on which we can build patient and community trust in the GPC, but GPC members also have strong regional collaborations between academic and health system informatics departments that are complemented by healthcare system and university governance (RC5). We are eager to participate in the national network. Modeled after existing national healthcare system collaborations (UHC), our low-cost infrastructure (RC14) and current regional policies to maintain patient and clinician privacy and confidentiality will align

with community-based practices and address organizational privacy sensitivities so that the GPC will allow for data sharing nationally, both within and outside of the network (RC7).

While the GPC is a new network, proven technologies based on two common EHR systems (Epic and Cerner) used at our sites, shared open source software, strong research infrastructures (e.g., CTSA programs, NCI-funded cancer centers), and experience with patient-centered outcomes research (PCOR) and comparative effectiveness research (CER) at each site will enable implementation and ensure sustainability of the GPC network. Most notably, the GPC will provide a blueprint for creating de novo highly functional research data networks to conduct pragmatic PCOR and CER trials required for a national learning healthcare system.

View Phase II<http://www.pcori.org//research-results/2015/greater-plains-collaborative>

View more about this project on PCORnet.org <http://pcornet.org/clinical-data-research-networks/cdrn4-university-of-kansas-medical-center-great-plains-collaborative/>.

More on this Project

Published Articles on the Results of this Project

ACTA ONCOLOGICA

Preferences and actual chemotherapy decision-making in the greater plains collaborative breast cancer study

Published Articles on this Project

JOURNAL OF THE AMERICAN MEDICAL INFORMATICS ASSOCIATION

The Greater Plains Collaborative: a PCORnet Clinical Research Data Network

Project Details

Principal Investigator

Lemuel Waitman, PhD

Project Status

PCORnet: Phase I completed; Phase II in progress

Project End Date

September 2015

Organization

University of Kansas Medical Center

Year Awarded

2013

State

Kansas

Funding Announcement

PCORnet: Clinical Data Research Networks (CDRN) Phase I

Project Budget

$6,429,697

Study Registration Information

HSRP20143467 <https://wwwcf.nlm.nih.gov/hsr_project/view_hsrproj_record.cfm?nlmunique_id=20143467>

Page Last Updated: November 13, 2017

© 2011-2018 Patient-Centered Outcomes Research Institute. All Rights Reserved.
